# Positive and negative regulation of the Fc**γ** receptor–stimulating activity of RNA-containing immune complexes by RNase

**DOI:** 10.1172/jci.insight.167799

**Published:** 2023-08-22

**Authors:** Ryota Naito, Koichiro Ohmura, Shuhei Higuchi, Wataru Nakai, Masako Kohyama, Tsuneyo Mimori, Akio Morinobu, Hisashi Arase

**Affiliations:** 1 Department of Rheumatology and Clinical Immunology, Kyoto University Graduate School of Medicine, Sakyo-ku, Kyoto, Japan.; 2 Laboratory of Immunochemistry, World Premier International (WPI) Immunology Frontier Research Center, and; 3 Department of Immunochemistry, Research Institute for Microbial Diseases, Osaka University, Osaka, Japan.; 4 Department of Rheumatology, Kobe City Medical Center General Hospital, Kobe, Japan.; 5 Center for Infectious Diseases for Education and Research (CiDER), and; 6 Center for Advanced Modalities and DDS (CAMaD), Osaka University, Osaka, Japan.

**Keywords:** Autoimmunity, Antigen processing, Autoimmune diseases, Rheumatology

## Abstract

The U1RNP complex, Ro/SSA, and La/SSB are major RNA-containing autoantigens. Immune complexes (ICs) composed of RNA-containing autoantigens and autoantibodies are suspected to be involved in the pathogenesis of some systemic autoimmune diseases. Therefore, RNase treatment, which degrades RNA in ICs, has been tested in clinical trials as a potential therapeutic agent. However, no studies to our knowledge have specifically evaluated the effect of RNase treatment on the Fcγ receptor–stimulating (FcγR-stimulating) activity of RNA-containing ICs. In this study, using a reporter system that specifically detects FcγR-stimulating capacity, we investigated the effect of RNase treatment on the FcγR-stimulating activity of RNA-containing ICs composed of autoantigens and autoantibodies from patients with systemic autoimmune diseases such as systemic lupus erythematosus. We found that RNase enhanced the FcγR-stimulating activity of Ro/SSA- and La/SSB-containing ICs, but attenuated that of the U1RNP complex–containing ICs. RNase decreased autoantibody binding to the U1RNP complex, but increased autoantibody binding to Ro/SSA and La/SSB. Our results suggest that RNase enhances FcγR activation by promoting the formation of ICs containing Ro/SSA or La/SSB. Our study provides insights into the pathophysiology of autoimmune diseases involving anti-Ro/SSA and anti-La/SSB autoantibodies, and into the therapeutic application of RNase treatment for systemic autoimmune diseases.

## Graphical abstract

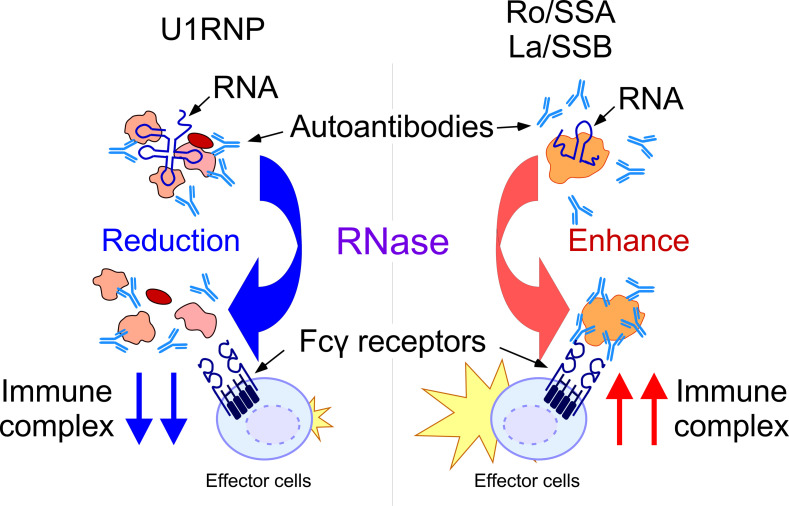

## Introduction

Systemic autoimmune diseases are caused by abnormal immune responses that damage various organs. Autoantibodies against nuclear components (antinuclear antibodies, ANAs) are often produced in systemic autoimmune diseases and are epidemiologically associated with specific clinical symptoms. Anti-U1RNP antibodies are one of the many types of ANAs and are associated with mixed connective tissue disease (MCTD), systemic lupus erythematosus (SLE), and some forms of systemic sclerosis (SSc). In addition, anti-Ro/SSA and anti-La/SSB (La) antibodies are other types of ANAs that are commonly detected in Sjögren’s syndrome (SS) and are used as diagnostic markers (1).

Several ANAs target RNA-containing protein complexes called ribonucleoproteins (RNPs), such as U1RNP complex and La (2, 3). The Ro/SSA antigen consists of 2 subunits, Ro52 and Ro60, and Ro60 binds directly to RNA (1, 4). Evidence has recently accumulated regarding the RNA-containing immune complex–induced (IC-induced) production of type I interferon (IFN) from plasmacytoid dendritic cells (pDCs) via Fcγ receptors (FcγRs) and RNA-sensing Toll-like receptors (TLRs), which are involved in the pathogenesis of various autoimmune diseases (5). Several reports showed that RNA-containing ICs induce IFN-α production by pDCs, and that RNase inhibits this IFN-α production (6–9). Based on these findings, a recombinant human RNase1 fused with the IgG1 Fc portion (RNase-Fc) that degrades RNA in ICs was expected to be effective in the treatment of SS patients possessing anti-Ro/SSA antibodies. However, IFN-induced gene expression was found to be increased in a clinical trial, although RNase1 treatment improved patient fatigue (10), indicating that RNase1 has the potential to stimulate autoimmune responses in SS. Thus, the role of RNase in the pathogenesis of systemic autoimmune diseases remains unclear.

Although the binding of anti-U1RNP antibodies to the U1RNP complex is reduced by RNase treatment (2), the major autoantibody epitopes on Ro60 and La are located in or near their RNA binding sites (3, 4, 11). Therefore, we hypothesized that the removal of RNA by RNase promotes the binding of anti-Ro60 and anti-La antibodies to Ro60 and La, respectively, resulting in the enhanced FcγR-stimulating activity of ICs containing Ro60 or La.

The FcγR-stimulating activity of RNA-containing ICs was previously evaluated by the activation of primary cells such as pDCs (6–8). However, pDCs express not only FcγRs but also various immune receptors such as TLRs. Therefore, it was impossible to specifically evaluate the FcγR-stimulating activity of RNA-containing ICs and the effect of RNase on such activity. We previously established FcγRIIIA-expressing nuclear factor of activated T cell–driven (NFAT-driven) green fluorescent protein–expressing (GFP-expressing) reporter cells (FcγRIIIA-reporter cells) that can specifically detect FcγRIIIA-stimulating activity (12–16). In this study, we investigated the effect of RNase on the FcγRIIIA-stimulating activity of ICs containing the U1RNP complex, Ro60, and La using the FcγRIIIA-reporter cells.

## Results

### Specific detection of the FcγRIIIA-stimulating activity of ICs using FcγRIIIA-reporter cells.

**Figure 1:**
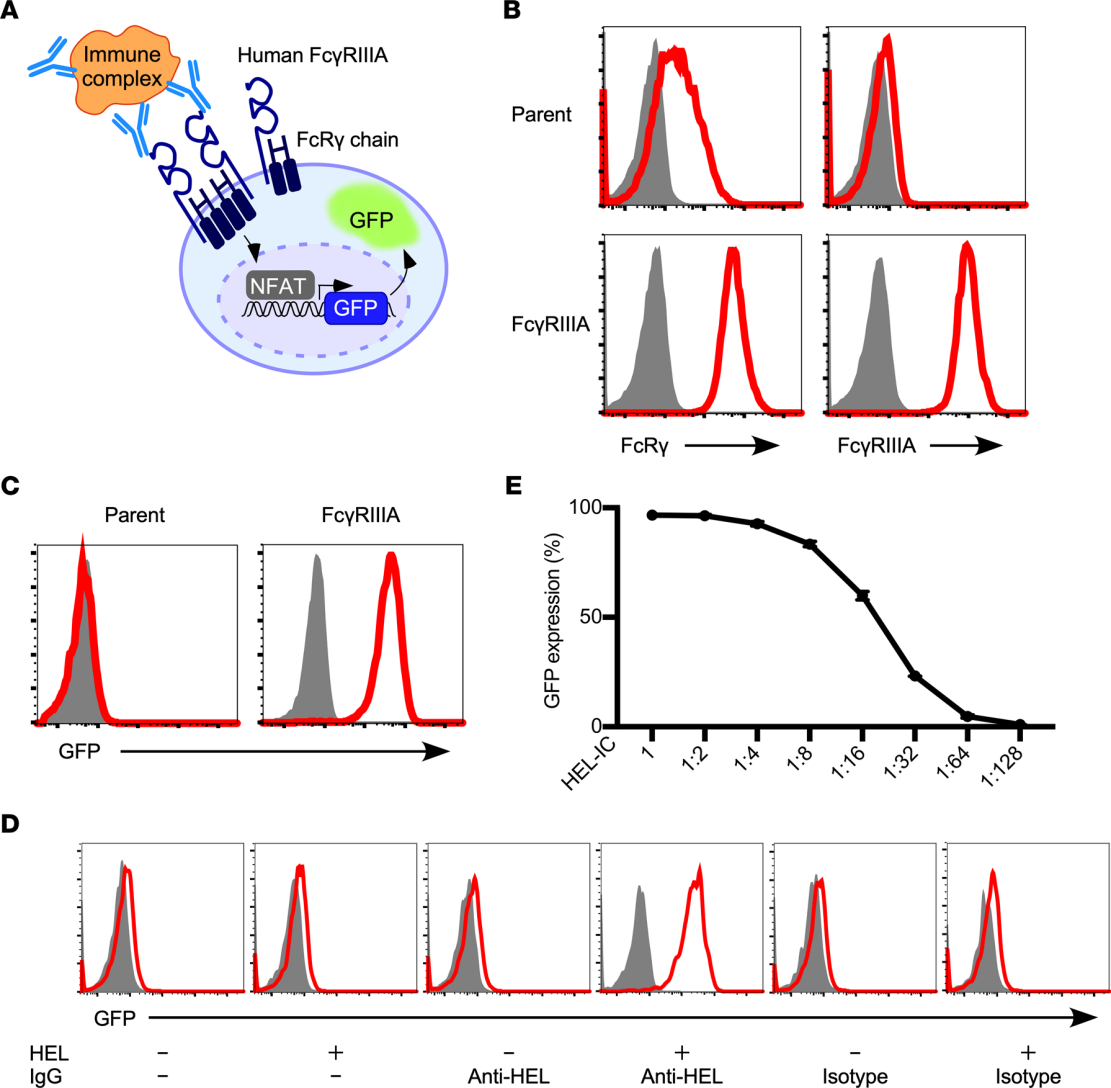
Quantitative measurement of FcγRIIIA-stimulating activity using NFAT-GFP reporter cells. (**A**) Schema of FcγRIIIA-transfected NFAT-GFP reporter cells (FcγRIIIA-reporter cells). (**B**) The reporter cells were stained with an anti-FLAG monoclonal antibody (mAb) and anti-FcγRIIIA mAb to examine FLAG-tagged FcRγ chain and FcγRIIIA expression. Shaded histograms represent isotype controls. (**C**) Activation of reporter cells by FcγRIIIA cross-linking stimulation by immobilized anti-FcγRIIIA mAb. Shaded histograms represent isotype controls. (**D**) FcγRIIIA stimulation by immune complexes (ICs) composed of HEL (50 ng/well) and anti-HEL human IgG1 (250 ng/well). Shaded histograms represent parental cells. (**E**) GFP expression of the FcγRIIIA reporter cells in relation to the serially 2-fold diluted HEL-IC (max: HEL 100 ng/well, anti-HEL IgG1 250 ng/well). FcγRIIIA, FcγRIIIA-reporter cells; parent, reporter cells lacking FcγRIIIA; isotype, human IgG1 isotype control.

To detect the FcγRIIIA-stimulating activity of ICs composed of ANAs and nuclear antigens, we generated FcγRIIIA-reporter cells in which FcγRIIIA-mediated signal transduction was detected by GFP expression (Figure 1A). The FcRγ chain, which associates with FcγRIIIA and mediates its activation signals, is expressed on the cell surface only in the presence of FcγRIIIA (17). Indeed, reporter cells expressing FLAG-tagged FcRγ alone were stained with anti-FLAG antibodies at low levels. In contrast, reporter cells expressing both FcRγ and FcγRIIIA were well stained with both anti-FcγRIIIA and anti-FLAG antibodies (Figure 1B). The FcγRIIIA-reporter cells expressed GFP upon stimulation with immobilized anti-FcγRIIIA monoclonal antibodies (Figure 1C). We next examined whether the FcγRIIIA-reporter cells are activated by ICs (Figure 1D). The FcγRIIIA-reporter cells were activated to express GFP in the presence of ICs generated by mixing hen egg lysozyme (HEL) with anti-HEL human IgG1 antibodies but not HEL or anti-HEL human IgG1 antibodies alone. Reporter cells expressing FcRγ chain alone were also not activated by ICs. FcγRIIIA-reporter cells were activated by ICs in a dose-dependent manner (Figure 1E). These results indicate that the FcγRIIIA-reporter cells specifically and quantitatively detect the FcγRIIIA-stimulating activity of soluble ICs.

**Figure 2:**
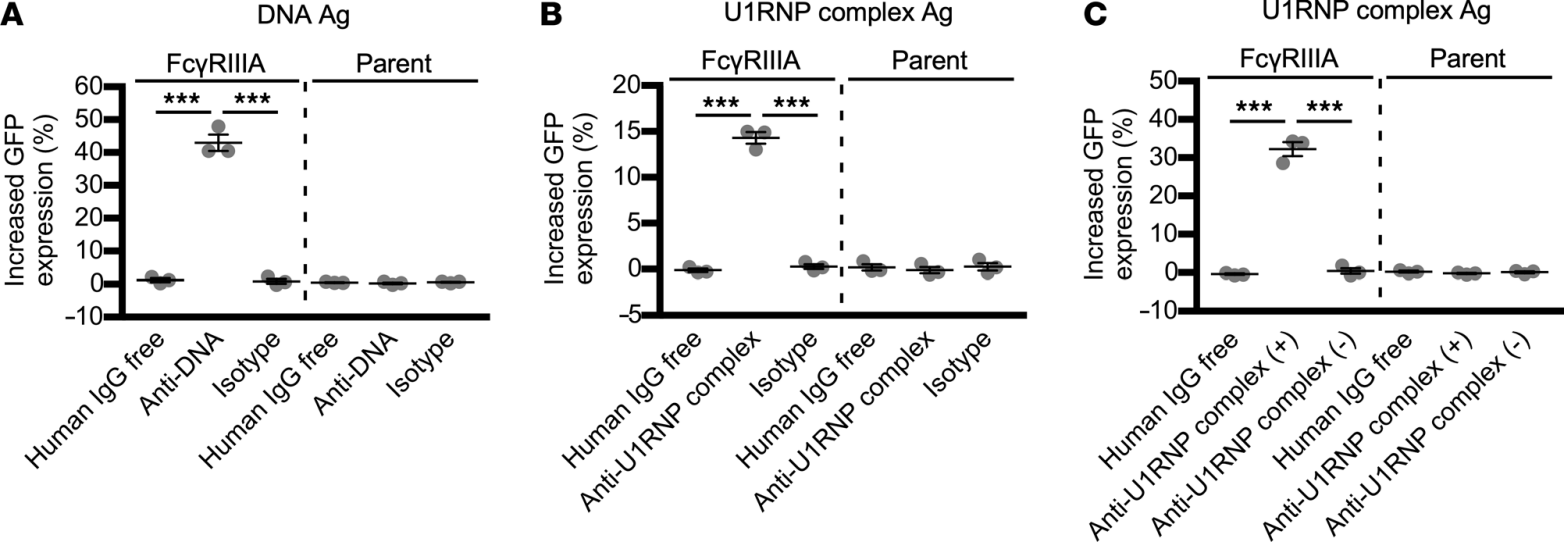
FcγRIIIA stimulation by ICs composed of nuclear antigens and antinuclear antibodies. The FcγRIIIA-reporter cells were incubated with mixtures of purified nuclear antigens and antinuclear antibodies as indicated below. (**A**) Purified DNA (1 μg/well) and anti-DNA monoclonal human IgG1 (500 ng/well). (**B**) U1RNP complex (50 ng/well) and anti–U1RNP complex monoclonal human IgG1 (250 ng/well 91E12 and 250 ng/well 113F3). (**C**) U1RNP complex (50 ng/well) and anti-U1RNP antibody–positive or –negative serum IgG obtained from patients with systemic lupus erythematosus (SLE) (500 ng/well). The vertical axis represents the GFP expression change (%) of the reporter cells relative to the antigen-free control. The significance of differences was determined by 1-way ANOVA and Šidák’s post hoc test. ***P < 0.001. See the Figure 1 legend for other definitions.

Next, we investigated the FcγR-stimulating activity of ICs composed of ANAs and nuclear antigens using the FcγRIIIA-reporter cells. ICs generated by mixing purified DNA with anti-DNA human IgG1 autoantibodies or by mixing the U1RNP complex with anti-U1RNP complex human IgG1 autoantibodies were found to stimulate the FcγRIIIA-reporter cells but not the reporter cells lacking FcγRIIIA (Figure 2, A and B). Control antibodies mixed with nuclear antigens did not stimulate the FcγRIIIA-reporter cells. Similarly, ICs generated by mixing the U1RNP complex with purified serum IgG from anti-U1RNP antibody–positive SLE patients stimulated the FcγRIIIA-reporter cells. However, U1RNP complex mixed with serum IgG from anti-U1RNP antibody–negative SLE patients did not stimulate the FcγRIIIA-reporter cells (Figure 2C and Table 1). These results indicate that the FcγRIIIA-reporter cells specifically detect the FcγRIIIA-stimulating activity of ICs composed of ANAs and nuclear antigens.

### RNase1 enhances the FcγRIIIA-stimulating activity of ICs composed of anti-Ro60 or anti-La antibodies.

**Figure 3:**
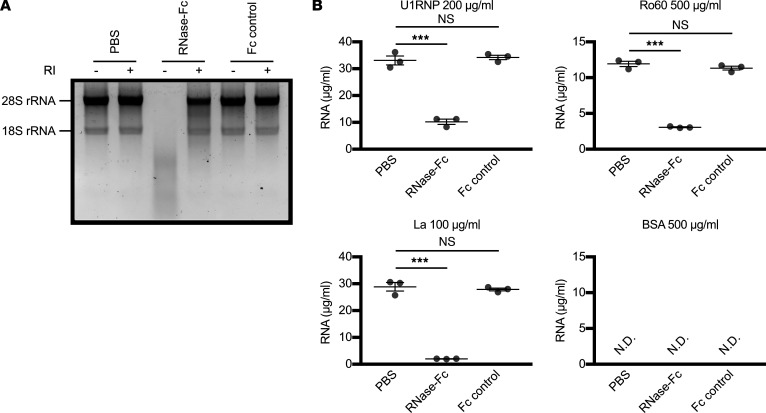
Degradation of RNA components of autoantigens by RNase-Fc. (**A**) Agarose electrophoresis of RNA purified from human cells treated with RNase-Fc. (**B**) The changes in RNA concentration of antigen solutions upon RNase-Fc treatment. For each antigen, the protein concentration is indicated at the top and the RNA concentration is plotted. The significance of differences was determined by 1-way ANOVA and Šidák’s post hoc test. ***P < 0.001. RI, RNase inhibitor; N.D., not detected (less than 1 μg/mL).

It has been reported that the binding of autoantibodies to the U1RNP complex is reduced by RNase, but the binding of autoantibodies to Ro60 and La is not reduced by RNase (2, 18–20). To investigate the effect of RNase on the FcγR-stimulating activity of RNA-containing ICs, we generated recombinant human RNase1 fused with the IgG1 Fc portion (RNase-Fc) in which the FcR binding sites were mutated, as RNase-Fc has also been used in previous clinical trials for SLE and SS (10, 21). RNase-Fc completely degraded RNA, and the RNase activity was blocked by an RNase inhibitor (Figure 3A).

**Figure 4:**
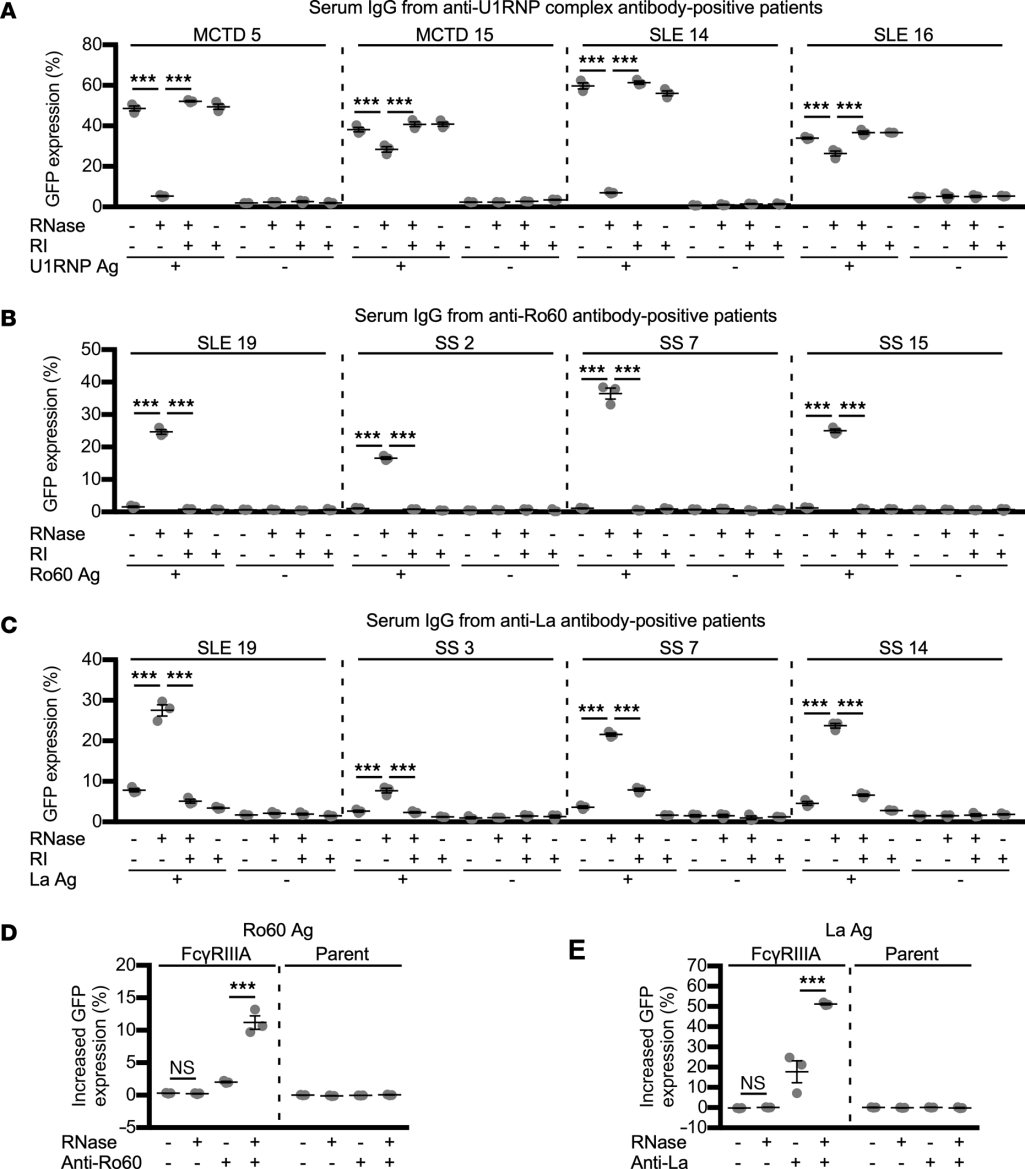
RNase treatment enhances the FcγRIIIA-stimulating activity of Ro60- and La-containing ICs. (**A**–**C**) The effect of RNase treatment on the FcγRIIIA-stimulating activity of mixtures of RNP antigens and serum IgG obtained from patients with systemic autoimmune diseases. (**A**) U1RNP complex and IgG from anti–U1RNP complex antibody–positive patients. (**B**) Ro60 and IgG from anti-Ro60 antibody–positive patients. (**C**) La and IgG from anti-La antibody–positive patients. Sample IDs are indicated at the top. (**D** and **E**) Effect of RNase on reporter cell activation by the mixture of Ro60 or La and IgG from patients with Sjögren’s syndrome (SS). In **D** and **E**, the vertical axis represents the GFP expression change (%) of the reporter cells relative to the Ro60- or La-free control. The significance of differences was determined by 1-way ANOVA and Šidák’s post hoc test. ***P < 0.001. RI, RNase inhibitor; MCTD, mixed connective tissue disease. See the Figure 1 legend for other definitions.

Next, we examined the effect of RNase on the FcγRIIIA-stimulating activity of ICs containing the U1RNP complex, Ro60, and La. As predicted, RNA was detected in the U1RNP complex, Ro60, and La, but not in the control protein, bovine serum albumin (BSA). Furthermore, the RNA in these antigens was depleted by treatment with RNase-Fc (Figure 3B). No FcγRIIIA-stimulating activity was observed for purified serum IgG obtained from patients in the absence of RNP antigen. Similarly, no stimulation of the FcγRIIIA-reporter cells was observed for RNase-Fc or RNase inhibitor (Figure 4, A–C). We also examined the effect of RNase on the FcγRIIIA-stimulating activity of ICs generated by mixing the U1RNP complex with serum IgG from 4 anti-U1RNP complex autoantibody–positive patients (Table 1). The FcγRIIIA-stimulating activity of the ICs generated by mixing U1RNP complex with serum IgG from anti-U1RNP autoantibody–positive MCTD or SLE patients was significantly decreased by RNase treatment, but the effect of RNase was inhibited by RNase inhibitor. The degree of reduction in the FcγRIIIA-stimulating activity of U1RNP-containing ICs by RNase treatment differed among patients, with an almost complete loss observed in 2 patients with MCTD or SLE and only a slight reduction observed in the other 2 patients (Figure 4A). These data indicate that RNase reduces the FcγRIIIA-stimulating activity of U1RNP-containing ICs.

**Figure 5:**
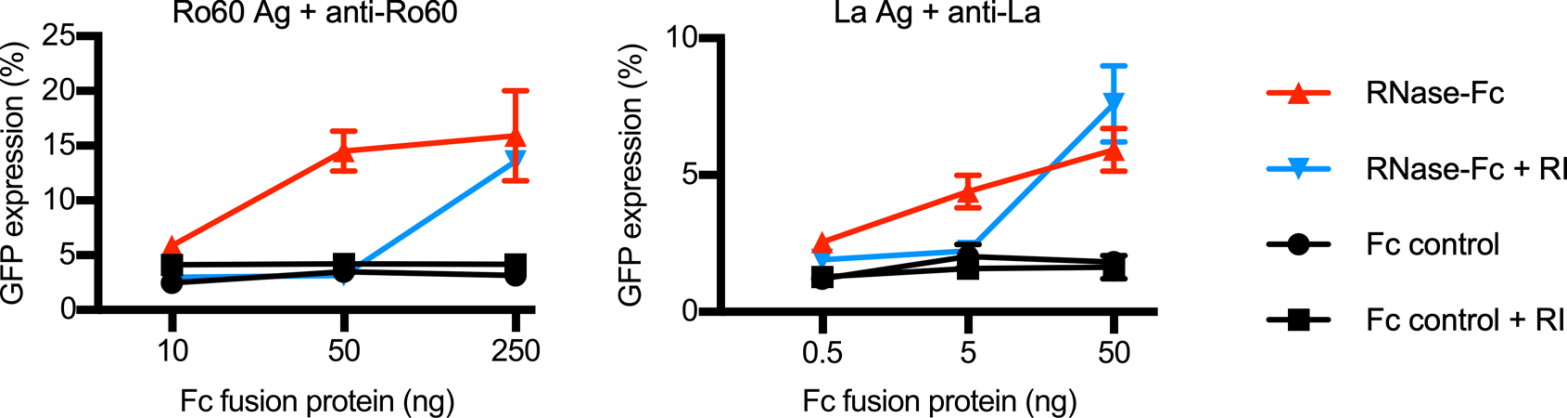
RNase enhances the FcγRIIIA-stimulating activity of Ro60- and La-containing ICs in a dose-dependent manner. Relationship between RNase dosage and FcγRIIIA-stimulating activity of Ro60- and La-containing ICs. Ro60 and La were incubated with RNase-Fc at the doses shown and added to FcγRIIIA-reporter cells in combination with serum IgG from anti-Ro60– or anti-La–positive SS patients, respectively. Red represents RNase-Fc only, blue represents the combination of RNase-Fc and RNase inhibitor (RI), and black represents Fc control.

**Figure 6:**
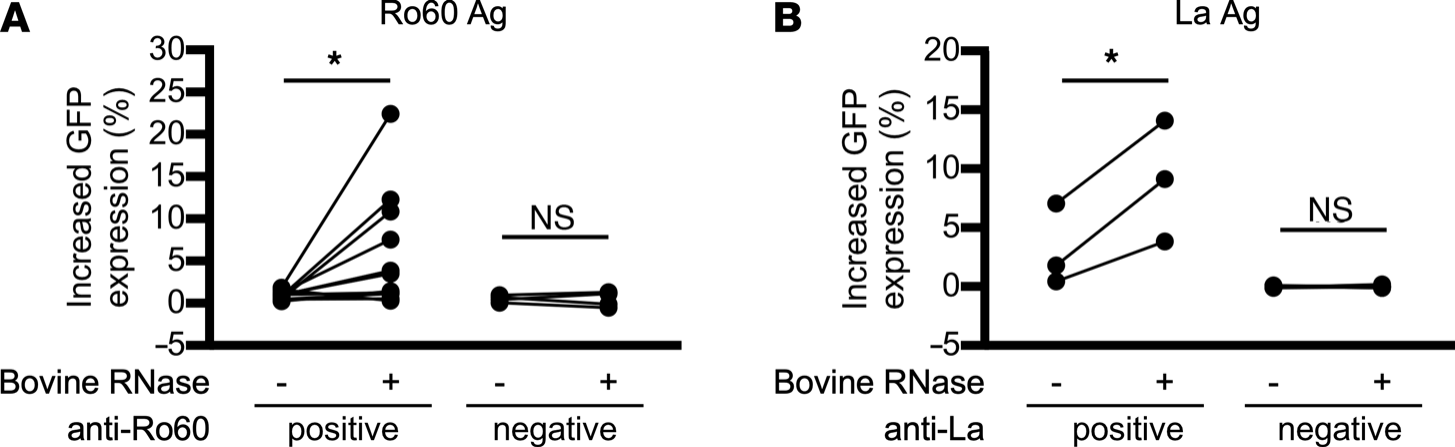
Bovine pancreatic RNase also enhances the FcγRIIIA-stimulating activity of Ro60- and La-containing ICs. The effect of bovine pancreatic RNase treatment on the FcγRIIIA-stimulating activity of Ro60- or La-containing ICs was examined. For RNase treatment, Ro60 or La and bovine pancreatic RNase were mixed in a PBS solution (50 ng of RNase per 10 ng of Ro60 or per 5 ng of La). Ro60 or La was then mixed with serum or plasma from 15 patients with SS and added to the FcγRIIIA-reporter cells. (**A**) The anti-Ro60 antibody–positive group (*n* = 11) and anti-Ro60 antibody–negative group (*n* = 4) are shown separately, and bovine pancreatic RNase–treated Ro60 and –untreated Ro60 were compared. (**B**) The anti-La antibody–positive group (*n* = 3) and anti-La antibody–negative group (*n* = 12) are shown separately, and bovine pancreatic RNase–treated La and –untreated La were compared. The vertical axis represents the change in GFP expression (%) relative to the Ro60- or La-free control for each condition, and each dot represents a single patient. The significance of differences was tested using a paired, 2-tailed *t* test. *P < 0.05.

Next, we examined the effect of RNase on the FcγRIIIA-stimulating activity of Ro60 mixed with serum IgG obtained from anti-Ro60 antibody–positive SLE or SS patients (Table 1). We found that the RNase-untreated Ro60 mixed with anti-Ro60 antibody–positive serum IgG showed almost no FcγRIIIA-stimulating activity. However, Ro60 mixed with anti-Ro60 antibody–positive serum IgG showed FcγRIIIA-stimulating activity upon RNase treatment, although the effect of RNase treatment differed among patients. The FcγRIIIA-stimulating activity observed in the presence of RNase was blocked by RNase inhibitor (Figure 4B). Next, we examined the effect of RNase on the FcγRIIIA-stimulating activity of La-containing ICs using serum IgG obtained from anti-La antibody–positive SLE or SS patients (Table 1). Similar to the ICs formed by Ro60 and anti-Ro60 antibodies, La mixed with serum IgG from anti-La antibody–positive SLE or SS patients did not stimulate the FcγRIIIA-reporter cells, but did stimulate the reporter cells upon RNase treatment. Furthermore, the FcγRIIIA-stimulating activity was abolished in the presence of RNase inhibitor (Figure 4C). RNase-treated Ro60 and La did not stimulate the reporter cells in the absence of anti-Ro60 or anti-La antibodies (Figure 4, D and E, and Table 1). The effects of RNase on the FcγRIIIA-stimulating activity of Ro60- and La-containing ICs occurred in a dose-dependent manner (Figure 5). These results suggest RNase treatment facilitated the binding of anti-Ro60 antibodies to Ro60 and anti-La antibodies to La to form ICs with FcγRIIIA-stimulating activity. Similar enhanced FcγRIIIA-stimulating activity was observed using bovine pancreatic RNase, indicating that the generation of ICs possessing FcγRIIIA-stimulating activity on RNase treatment in the presence of anti-Ro60 and anti-La antibodies was caused by the degradation of RNA bound to Ro60 and La (Figure 6, A and B).

**Figure 7:**
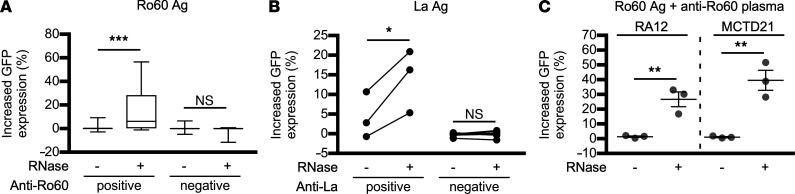
Enhancement of the FcγRIIIA-stimulating activity of Ro60 and La by RNase requires anti-Ro60 and anti-La antibodies, respectively. The effect of RNase on the FcγRIIIA-stimulating activity of Ro60 or La mixed with the serum or plasma from patients with systemic autoimmune diseases and healthy donors was examined. (**A**) For Ro60, 174 samples were examined and are shown separately as the anti-Ro60 antibody–positive group (*n* = 42) and –negative group (*n* = 132). (**B**) For La, 15 patients with SS were examined and are shown separately as the anti-La antibody–positive SS group (*n* = 3) and –negative SS group (*n* = 12). (**C**) The effect of RNase on the FcγRIIIA-stimulating activity of Ro60 mixed with anti-Ro60 antibody–containing plasma from a patient with rheumatoid arthritis (RA) or MCTD. In **A**, the median, 25th percentile, 75th percentile (box), and range (whiskers) are shown. In **B**, each dot represents 1 patient. In **C**, the results of 3 independent experiments with plasma from 1 patient are plotted. In **A**–**C**, the vertical axis represents the change in GFP expression (%) of reporter cells relative to the Ro60- or La-free control for each condition. The significance of differences was determined by paired, 2-tailed *t* test in **A** and **B**, and by 2-tailed Student’s *t* test in **C**. *P < 0.05, **P < 0.01, ***P < 0.001.

The effect of RNase treatment on the FcγRIIIA-stimulating activity of Ro60- and La-containing ICs was examined in large numbers of serum and plasma samples (Table 2). As expected, we found that RNase-treated Ro60 and La enhanced FcγRIIIA-stimulating activity only in the presence of anti-Ro60 and anti-La antibodies, respectively (Figure 7, A and B). These results confirm that RNase treatment promotes the formation of Ro60- and La-containing ICs with FcγRIIIA-stimulating activity. Furthermore, the enhancement of the FcγRIIIA-stimulating activity of Ro60-containing ICs by RNase treatment was observed in samples from patients with MCTD, RA, SLE, and SS, and the enhancement of the FcγRIIIA-stimulating activity of La-containing ICs by RNase treatment was observed in samples from patients with SLE and SS (Figure 4, B and C, and Figure 7C). These results suggest that RNase increases the FcγRIIIA-stimulating activity of Ro60-containing and La-containing ICs in various systemic autoimmune diseases.

### RNase1 enhances the binding of autoantibodies to Ro60 and La antigens.

**Figure 8:**
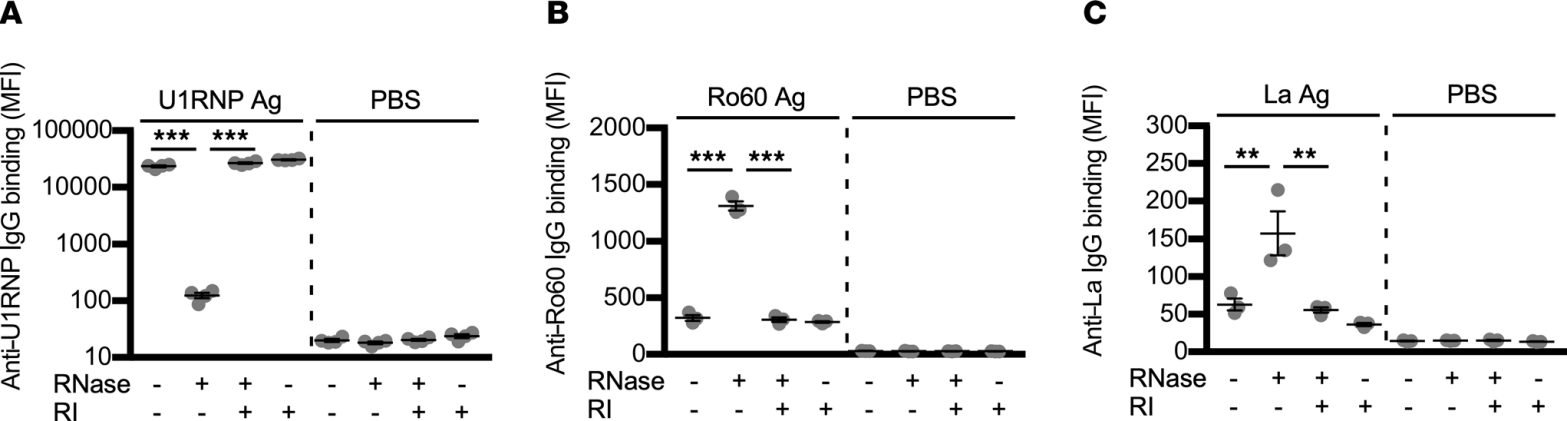
RNase increases the binding of anti-Ro60 antibodies to Ro60 and anti-La antibodies to La. As indicated below, RNase-treated or -untreated RNP antigens captured on autoantibody-coupled beads were detected by biotinylated serum IgG from patients with systemic autoimmune diseases and allophycocyanin-labeled streptavidin. (**A**) U1RNP complex captured on anti-U1RNP complex monoclonal antibody–coupled beads was detected by serum IgG from anti-U1RNP complex antibody–positive patients (ID: MCTD5 and SLE14). (**B**) Ro60 captured on anti-Ro60 rabbit antibody–coupled beads was detected by serum IgG from anti-Ro60 antibody–positive patients (ID: SLE19 and SS15). (**C**) La captured on anti-La rabbit antibody–coupled beads was detected by serum IgG from anti-La antibody–positive patients (ID: SLE19 and SS3). The significance of differences was determined by 1-way ANOVA and Šidák’s post hoc test. **P < 0.01; ***P < 0.001.

To investigate the mechanisms by which RNase affects the FcγRIIIA-stimulating activity of RNA-containing ICs, we examined the effect of RNase on the binding of autoantibodies to RNP antigens. U1RNP complex, Ro60, and La antigens were captured on latex beads coupled with anti–U1RNP complex antibody, anti-Ro60 rabbit antibody, or anti-La antibody, respectively, and serum autoantibody binding to the captured RNP antigens was analyzed by flow cytometry. The binding of the serum IgG from anti-U1RNP complex antibody–positive MCTD or SLE patients to the U1RNP complex was significantly decreased by RNase treatment. The effect of RNase treatment was inhibited by RNase inhibitor (Figure 8A). These results suggest that RNA is required for the binding of anti-U1RNP complex antibodies to the U1RNP complex.

In contrast, serum IgG from anti-Ro60 antibody–positive SLE or SS patients bound to the Ro60 antigen at low levels, but the binding was significantly enhanced by RNase treatment. The enhanced autoantibody binding to Ro60 by RNase treatment was negated in the presence of RNase inhibitor (Figure 8B). Similarly, binding of serum IgG from anti-La antibody–positive SLE or SS patients to La antigen was significantly enhanced by RNase treatment, and RNase inhibitor blocked the enhancing effect of RNase treatment (Figure 8C). RNase inhibitor did not affect autoantibody binding to RNase-untreated Ro60 or La antigens (Figure 8, B and C). These results suggest that RNase treatment exposes autoantibody epitopes on Ro60 and La antigens, which promotes autoantibody binding to these RNA-binding antigens.

### RNase activities in patients with systemic autoimmune diseases.

**Figure 9:**
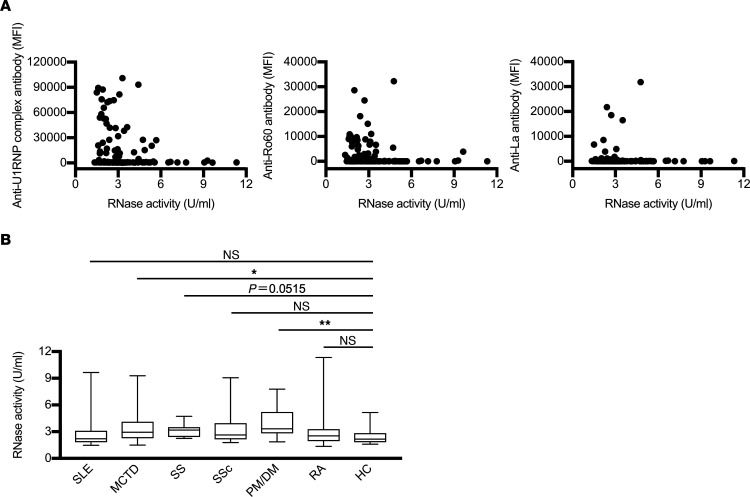
Serum and plasma RNase activity is increased in systemic autoimmune diseases. RNase activity in serum or plasma of patients with systemic autoimmune diseases and healthy controls (HC) was measured. (**A**) Scatter plots showing RNase activity on the horizontal axis and autoantibody titers on the vertical axis for serum or plasma from patients with systemic autoimmune diseases (*n* = 124). Anti–U1RNP complex, anti-Ro60, and anti-La antibody are shown separately. (**B**) RNase activity in serum or plasma of SLE (*n* = 30), MCTD (*n* = 27), SS (*n* = 15), systemic sclerosis (SSc) (*n* = 16), polymyositis and dermatomyositis (PM/DM) (*n* = 17), RA (*n* = 19), and HC (*n* = 50) are shown separately. The significance of differences was determined by Kruskal-Wallis test and Dunn’s post hoc test. *P < 0.05, **P < 0.01.

Finally, to address the involvement of RNase in the pathogenesis of systemic autoimmune diseases, we analyzed RNase activities in blood. However, there was no clear correlation between RNase activity and anti–U1RNP complex, anti-Ro60, or anti-La autoantibody titers (Figure 9A). On the other hand, RNase activity was slightly elevated in the patients with systemic autoimmune diseases compared with healthy control individuals. RNase activity, in particular, was significantly elevated in the patients with polymyositis and dermatomyositis (PM/DM) and MCTD. RNase activity was also high, but not significantly so, in patients with SS (Figure 9B). These results suggest that RNase activity is elevated in some patients with systemic autoimmune diseases, although there was no significant correlation with autoantibody titers.

## Discussion

RNase decreased the binding of autoantibodies to the U1RNP complex as well as the FcγRIIIA-stimulating activity of U1RNP complex–containing ICs. The U1RNP complex is composed of the U1-specific proteins U1-70k, U1-A, and U1-C and common Sm proteins, with U1-RNA as the core (2). U1-RNA is important for the binding of anti-U1RNP antibodies to the U1RNP complex, as U1-RNA serves as the core for the U1RNP complex and forms higher-order structural autoantibody epitopes on the U1RNP complex (2, 22). RNase appears to reduce the binding of anti-U1RNP antibodies by degrading the U1-RNA in the U1RNP complex, thereby dispersing U1-specific proteins and disrupting the higher-order structural autoantibody epitopes. The differences in the degree of reduction in the FcγRIIIA-stimulating activity of U1RNP complex–containing IC by RNase among our samples may be due to differences in the titers of the autoantibodies recognizing the U1-RNA–independent epitopes on the U1RNP complex.

In contrast, RNase facilitated the binding of anti-Ro60 and anti-La antibodies to Ro60 and La, respectively, and enhanced the FcγRIIIA-stimulating activity of Ro60- or La-containing ICs. Ro60 binds to the members of a class of noncoding RNAs (ncRNAs) called Y RNAs, as well as to misfolded and aberrant ncRNAs. Moreover, La binds to the ends of all nascent transcripts of RNA polymerase III, including Y RNAs (23). Epitope mapping studies using short peptides and recombinant protein fragments of Ro60 and La have suggested that the major autoantibody epitopes on these autoantigens are localized around their RNA binding sites (3, 4, 11). However, it is possible that the autoantibody epitopes predicted using short peptides or recombinant protein fragments do not represent the epitopes dependent on higher-order structures. Indeed, the epitopes on Ro60 are dependent on higher-order structures, and some anti-Ro60 antibodies bind to native Ro60 but not to recombinant Ro60 (4, 11, 24). Furthermore, it was unclear whether exposure of the cryptic autoantibody epitopes on Ro60 and La by removal of RNA would affect the subsequent immune response. We demonstrated that RNase treatment enhances the binding of autoantibodies to native Ro60 and La antigens, suggesting that RNA masks the major autoantibody epitopes on native Ro60 and La and blocks autoantibody binding. On the other hand, autoantibodies also bound to RNA-containing Ro60 and La antigens at low levels. As some epitopes outside the RNA-binding sites of Ro60 and La have also been reported (3), our results suggest that anti-Ro60 and anti-La antibodies that bind outside the RNA-binding sites can be detected even with RNase-untreated Ro60 and La antigens. Furthermore, we demonstrated that removal of RNA from Ro60 and La by RNase enhanced the FcγRIIIA-stimulating activity of their ICs.

RNA-containing ICs are involved in RNA-sensing TLR-mediated immune cell activation. On the other hand, FcγR-mediated effector cell responses such as antibody-dependent cellular cytotoxicity, phagocytosis, and cytokine release, do not always require signal transduction through the RNA-sensing TLRs (15, 17, 25). Enhanced phagocytosis of antigens induced by FcγR stimulation increases antigen presentation to T cells (26). Furthermore, FcγR stimulation has been reported to synergistically enhance cytokine production by effector cells in response to the stimulation of pattern recognition receptors, including RNA-sensing TLRs, and cytokine receptors (27–29). Collectively, our results suggest that RNase not only suppresses the direct stimulation of TLRs by RNA-containing ICs, but also has the potential to promote immune responses through FcγRs in systemic autoimmune diseases associated with anti-Ro60 and anti-La antibodies.

RNase production is reported to be enhanced at sites of inflammation and tissue injury. The RNase A family is a family of secreted RNases, currently consisting of RNase1 to RNase13, that are expressed in various organs and immune cells, have immunomodulatory and antimicrobial activities, and are secreted in response to tissue inflammation or injury (30). RNase1 is released from vascular endothelial cells at sites of acute inflammation (31). The salivary gland epithelium of SS forms an important niche for antigen-driven affinity maturation and persistence of anti-Ro60 and anti-La antibodies (32, 33), and RNase7 production was shown to be enhanced in salivary glands with lymphocytic infiltrates in SS (34). RNase T2 is upregulated in response to tissue injury and functions as an intracellular and extracellular RNase (35). Viral infections and increased expression of IFN-induced genes in systemic autoimmune diseases may induce RNase-L activation (5, 36). Increased RNase production was observed in some patients with systemic autoimmune diseases. However, RNase activity was not significantly associated with anti–U1RNP complex, anti-Ro60, or anti-La antibody titers, although it is possible that RNase activity in serum may not reflect local RNase activity in specific tissues. RNase produced upon inflammation or tissue damage might locally promote anti-Ro60 and anti-La autoantibodies to form ICs with Ro60 and La released from necrotic or apoptotic cells to cause the FcγR-mediated inflammation. Indeed, the epitope of the anti-Ro60 antibody produced in the earliest stage of SLE is located in the RNA-binding site of the Ro60 antigen (4). Therefore, local RNase production might be involved in autoantibody production by exposing cryptic epitopes on Ro60.

The administration of RNase has been widely investigated for application to the treatment of various inflammatory and ischemic diseases (31), and RNase-Fc is one of candidates for the treatment of systemic autoimmune diseases (5). However, a clinical trial of RNase treatment in anti-Ro antibody–positive SS patients showed the increased IFN-induced gene expression, with the mechanism underlying the enhanced immune response remaining unclear (10). Our results suggest that RNase exacerbates immune responses in the presence of anti-Ro60 and anti-La antibodies by enhancing the FcR-stimulating activity of ICs, and that caution should be exercised in the clinical application of RNase therapy. RNase treatment might be effective in patients with anti–U1RNP complex antibodies but without anti-Ro60 and anti-La antibodies. Further studies are needed to select appropriate patients suitable for RNase therapy, both to ensure safety and enhance therapeutic efficacy.

A limitation of this study is that we addressed only 3 representative RNA-associated antigens. As other autoantibodies targeting RNA-associated antigens such as aminoacyl-tRNA synthetases also appear (1), other RNA-associated antigens should be investigated to provide a better understanding of the role of RNase in the pathogenesis of systemic autoimmune diseases. In addition, we specifically analyzed the effect of RNase on FcγRIIIA-stimulating activity of RNA-containing ICs. Although activating FcγRs are the major targets for ICs, most immune cells possessing activating FcγRs also express other types of immune receptors, such as inhibitory FcγRIIB and RNA-sensing TLRs. Therefore, the effects of RNase on ICs would be modulated by these receptors. Further studies are required to clarify the exact function of RNase on RNA-containing ICs in vivo (5, 17).

In summary, RNase reduced the FcγRIIIA-stimulating activity of U1RNP complex–containing ICs, but enhanced that of both Ro60- and La-containing ICs. These findings suggest that RNase may be involved in promoting autoimmune responses to Ro60 and La. Further studies are needed to elucidate the role of RNase in systemic autoimmune diseases.

## Methods

### Human samples.

Serum and plasma samples from 124 patients with systemic autoimmune diseases were obtained from Kyoto University. According to the classification criteria, 27 patients were classified as MCTD (37), 17 patients as “probable” or “definite” PM/DM (38), 19 patients as rheumatoid arthritis (RA) (39), 30 patients as SLE (40, 41), 15 patients as SS (42), and 16 patients as SSc (43). Patients meeting the MCTD classification criteria were classified as MCTD, even when the patients satisfied the classification criteria of other autoimmune diseases. All patients met only one classification criterion, except for the PM/DM, SLE, or SSc criteria in MCTD patients. CORE Kit (George King Bio-Medical) samples were used as healthy controls. IgG samples were purified from patients from whom sufficient serum or plasma samples were obtained, using recombinant protein A–Sepharose (GE Healthcare).

### Plasmids.

For production of recombinant human RNase-Fc, a pCAGGS expression vector encoding a mouse CD150 leader sequence at the N-terminus and the Fc portion of human IgG1 at the C-terminus (mutated to reduce binding to FcRs) was used as described previously (44). The full-length complementary DNA (cDNA) of human *RNASE1* (accession no. NM_198234.3) was obtained by PCR amplification from cDNA prepared from human pancreatic total RNA (Clontech). A pCAGGS vector encoding the human IgG1 Fc portion without human RNase1 (Fc control) was also generated. Sequences of all constructs were confirmed by DNA sequencing (ABI 3130xl DNA Sequencer; Applied Biosciences). The expression vectors encoding anti-DNA human IgG1 (71F12) and anti–U1RNP complex human IgG1 (91E12 and 113F3) were provided by Hitoshi Kikutani (Osaka University, Osaka, Japan) (45). Plasmids for anti-HEL IgG1 (HyHEL10) and human *FCGR3A 176V* genotype (accession no. NM_000569.7) were used as previously described (15, 46).

### Cell lines.

As previously described (15), FcγRIIIA-reporter cells were generated by retroviral transfection of full-length FcγRIIIA into a mouse T cell hybridoma stably transfected with the NFAT-GFP reporter gene and the FLAG-tagged FcRγ gene (parental cell). Expi293F cells were purchased from Thermo Fisher Scientific. These cells were routinely examined for mycoplasma contamination.

### Antibodies and recombinant proteins.

Anti-FcγRIIIA monoclonal antibody (3G8; BioLegend), anti-FLAG monoclonal antibody (L5; BioLegend), anti-Ro60 rabbit polyclonal antibody (HPA002835; Sigma-Aldrich), anti-La rabbit polyclonal antibody (AV40461; Sigma-Aldrich), bovine pancreatic RNase (Roche), and RNase inhibitor (RNasin; Promega) were purchased as indicated. Anti-DNA human IgG1 (71F12), anti–U1RNP complex human IgG1 (91F12 and 113F3), anti-HEL human IgG1 (HyHEL10), RNase-Fc, and Fc control were obtained by transfection of each expression vector into Expi293F cells (Gxpress 293 Transfection Kit; Gmep) and purification from the culture supernatant using recombinant protein A–Sepharose (GE Healthcare).

### Purified DNA and RNA.

DNA and RNA were purified from Expi293F cells using purification kits (Wizard SV Genomic DNA Purification System, Promega; RNeasy Mini Kit, Qiagen), and suspended in PBS.

### Purified nuclear antigens.

Bovine and/or rabbit U1RNP complex (SRC-3000; ImmunoVision), Ro60 from calf (ATR02; AROTEC), and La from calf and/or rabbit (SSB-3000; ImmunoVision) origins were purchased.

### Measurement of autoantibody titers in serum and plasma samples.

U1RNP complex–, Ro60-, or La-coupled Aldehyde/Sulfate Latex Beads (Life Technologies) were incubated with serum/plasma samples (diluted 1:1000) followed by allophycocyanin-conjugated (APC-conjugated) anti–human IgG (H+L) (Jackson ImmunoResearch), and the levels of IgG bound to the beads were analyzed using FACSVerse (Becton Dickinson). The positive cutoff values were defined as relative to the 99th percentile value of 50 healthy control samples, greater than 2-fold for anti-U1RNP complex antibody, greater than 20-fold for anti-Ro60 antibody, and greater than 30-fold for anti-La antibody.

### Reporter assay.

The FcγRIIIA-reporter cells were suspended in RPMI1640 medium with 10% FBS, seeded into 384-well culture plates (Greiner Bio-One) at 2500 cells per well, and incubated overnight or for 2 nights with antigen and IgG or serum/plasma samples. FcγRIIIA-stimulating activity was evaluated by the proportion of reporter cells expressing GFP using FACSVerse (Becton Dickinson). Stimulation of reporter cells with immobilized antibodies was performed as previously described (15). For RNase treatment, U1RNP complex, Ro60, or La and RNase were mixed in PBS solution, incubated at 37°C for 1 hour, and then added to plated reporter cells with IgG or serum/plasma samples. RNase-Fc (or Fc control) was added at 1 ng per 10 ng of U1RNP complex, 5 ng per 10 ng of Ro60, and 1 ng per 10 ng of La, respectively. When used, RNase inhibitor was added at 1 U per 5 μg of RNase-Fc. U1RNP complex was added at 50 ng per well, Ro60 at 100 ng per well, and La at 50 ng per well, respectively. Anti-U1RNP–positive IgG was added at 500 ng per well, anti-Ro60–positive IgG at 1 μg per well, anti-La–positive IgG at 1 μg per well, and serum/plasma samples at 25 nL per well.

### Assay of RNase activity by nondenaturing agarose gel electrophoresis.

One microgram of purified RNA was incubated with 0.5 ng of RNase-Fc with or without 20 U of RNase inhibitor at 37°C for 30 minutes, and then heated at 70°C for 10 minutes, electrophoresed in a 1% agarose gel, stained with SYBR Safe DNA Gel Stain (Thermo Fisher Scientific), and subsequently imaged (ChemiDoc Touch; Bio-Rad).

### Measurement of RNA concentration in antigen solutions.

U1RNP complex, Ro60, La, and BSA were suspended in PBS at 200, 500, 100, and 500 μg/mL, respectively, with or without 50 μg/mL RNase-Fc (or Fc control). After incubation at 37°C for 1 hour, the RNA concentration of these antigen solutions was measured using Qubit RNA BR Assay Kit (Thermo Fisher Scientific).

### Measurement of RNase activity in serum and plasma samples.

Serum and plasma samples were diluted 3000-fold in RNase-free water, and their RNase activity was measured using the RNaseAlert QC System (Thermo Fisher Scientific). RNase activity was determined by a calibration curve prepared using serially diluted RNase A.

### Detection of the immunoprecipitation of RNase-treated antigens by flow cytometry.

U1RNP complex, Ro60, and La were each suspended in PBS, and RNase-Fc (or Fc control) was added at 10 ng per 10 ng of U1RNP complex, 50 ng per 10 ng of Ro60, and 10 ng per 10 ng of La and incubated at 37°C for 1 hour. RNase inhibitor was, if used, added at 1 U per 5 μg of RNase-Fc. The RNase-treated U1RNP complex, Ro60, and La were then incubated with anti-U1RNP complex monoclonal antibody (91E12)–, anti-Ro60 rabbit antibody–, or anti-La rabbit antibody–coupled latex beads (Aldehyde/Sulfate Latex Beads; Life Technologies) at 4°C for 20 minutes. Thereafter, the latex beads were washed and again incubated with biotinylated serum IgG from patients prepared by EZ-Link Sulfo-NHS-LC-Biotin (Thermo Fisher Scientific) at 4°C for 20 minutes. The beads were then washed, stained with APC-streptavidin (Jackson ImmunoResearch), and analyzed with FACSVerse (Becton Dickinson).

### Statistics.

Two-tailed Mann-Whitney *U* test, Student’s *t* test, paired *t* test, 1-way ANOVA with Šidák’s post hoc test, or Kruskal-Wallis test with Dunn’s post hoc test was used to determine the significance of differences. Adjusted *P* values of less than 0.05 were considered statistically significant. Calculations and graphing were performed using Prism 7.0 software (GraphPad Software). Significance levels were assigned as follows: NS, not significant, *P < 0.05, **P < 0.01, ***P < 0.001. Unless otherwise indicated, the values in the figures are the mean ± SEM of triplicate experiments. All results in the figures are representative of at least 3 independent experiments.

### Study approval.

Protocols for the collection and use of serum and plasma samples were approved by the Institutional Review Boards (IRBs) of Kyoto University (G1006-13, R1540-6) and Osaka University (IFReC imm-4-2, 2020-19). Written informed consent was obtained from all participants in accordance with the Declaration of Helsinki and the relevant guidelines of the respective IRBs.

## Author contributions

RN, KO, and HA conceived of the study. RN and SH developed the protocol. RN and SH did the literature search. RN and SH appraised study quality, and extracted and analyzed the data. All authors contributed to the data interpretation. All authors contributed to the preparation of the manuscript, approved the final submitted version, and agreed to be listed as authors. HA is responsible for the overall content as the guarantor.

## Supplementary Material

Supporting data values

## Figures and Tables

**Table 2:**
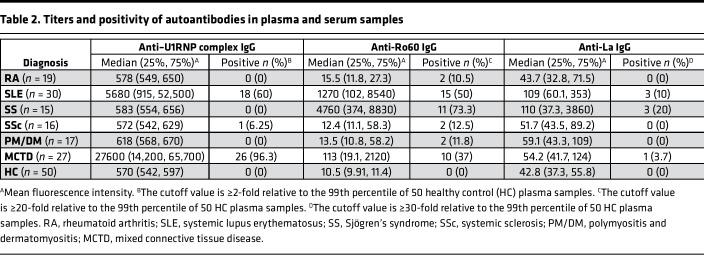
Titers and positivity of autoantibodies in plasma and serum samples

**Table 1:**
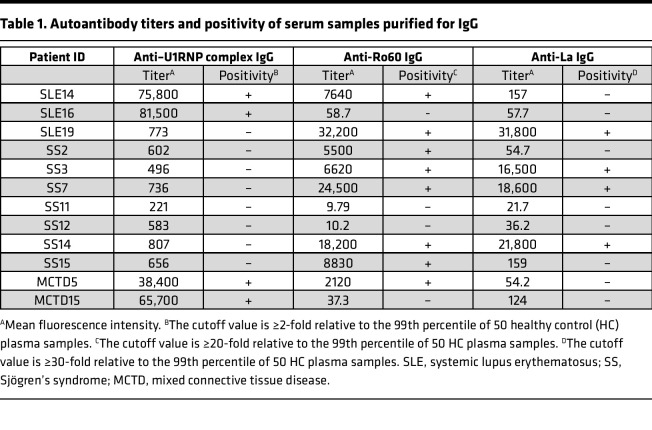
Autoantibody titers and positivity of serum samples purified for IgG

## Version 1 07/11/2023

In-Press Preview

## Version 2 08/22/2023

Electronic publication
